# Identification of Therapeutic Potential of Hydroxychavicol Against Alzheimer's Disease: An Integrated Network Pharmacology, Molecular Docking, and Dynamic Simulation Study

**DOI:** 10.1155/jare/7062203

**Published:** 2025-03-25

**Authors:** Priyank Upadhayay, Saurabh K. Sinha, Neeraj Kumar, Shashi Kant Singh, Preet Jain, Sunita Panchawat, Nitish Rai

**Affiliations:** ^1^Department of Biotechnology, Mohanlal Sukhadia University, Udaipur, India; ^2^Department of Pharmaceutical Sciences, Mohanlal Sukhadia University, Udaipur, Rajasthan, India; ^3^Department of Pharmaceutical Chemistry, B.N. College of Pharmacy, Udaipur, Rajasthan, India; ^4^Faculty of Pharmaceutical Sciences, Mahayogi Gorakhnath University, Gorakhpur, Uttar Pradesh, India; ^5^Department of Prosthodontics, R.R. Dental College & Hospital, Udaipur, Rajasthan, India; ^6^Department of Zoology, University of Lucknow, Lucknow, Uttar Pradesh, India

**Keywords:** Alzheimer's disease, hydroxychavicol, molecular docking, molecular dynamics, network pharmacology

## Abstract

Alzheimer's disease (AD) is a commonly occurring neurodegenerative disease in elderly and it is a leading cause of dementia worldwide. Hydroxychavicol (HC), a major phenolic component of *Piper betle,* has prominent anti-inflammatory and antioxidant properties, and studies have found its role in cognition improvement. Here is a systematic approach to deciphering the potential protein targets of HC in AD through network pharmacology and validation from molecular docking and dynamics simulation study. First, the druglikeliness of HC was predicted using the SwissADME analysis, which showed significant druglikeliness. A total of 88 possible target genes between HC and AD were obtained from the Swiss Target Prediction, HIT Version 2, DisGeNET, and GeneCards database. The pathway analysis was carried out using the STRING database which showed several genes including COMT, HSP90AA1, and GAPDH as the top hub genes on the basis of degree. GO and KEGG analyses demonstrated that the core targets were mainly involved in cAMP, PI3K/AkT, HIF1, Rap1, and Calcium signaling pathways. The molecular docking of HC with top hub genes resulted in the highest binding of HC with COMT (−8.9 kcal/mol), GAPDH (−6.7 kcal/mol), and HSP90AA1 (−6.5 kcal/mol) that showed stable binding in the molecular dynamics simulation study. COMT regulates the dopamine levels in the prefrontal cortex and impairment of the COMT is associated with the rapid progression of AD. HSP90, a ubiquitous molecular chaperone, is involved in regulating tau metabolism and Aβ processing and found to be downregulated in AD. GAPDH has been reported as the disease-susceptible gene in AD and its interaction with amyloid precursor protein and NFTs has also been reported. These findings suggest that HC is a promising therapeutic candidate, targeting multiple AD-related pathways, warranting further investigation into its molecular mechanisms and potential for clinical application.

## 1. Introduction

Alzheimer's disease (AD) is the most common neurodegenerative disease and the leading cause of dementia worldwide, typically occurring in the elderly and near elderly and rarely in young (early onset). It is characterized by progressive neuronal loss, cognitive impairment, and memory degeneration. The worldwide cases of dementia and associated diseases have increased to over 55 million in the year 2020 and are expected to increase to 82 million by the year 2030 and 152 million in 2050 [1]. The pathophysiological understanding of AD has improved in recent years and various important underlying mechanisms are being targeted for drug development [2]. There are two types of approved drugs for the treatment of AD, which include acetylcholinesterase inhibitors (donepezil, rivastigmine, and galantamine) and N-methyl D-aspartate (NMDA) receptor blockers (memantine) [3]. These drugs provide only symptomatic relief and do not cure the disease. Recent studies have documented the increasing research on immunotherapies for the treatment of AD, and many anti-Aβ antibodies such as aducanumab, lecanemab, and donanemab are in clinical trials. Recently, the monoclonal antibody lecanemab (Leqembi) received FDA approval for the treatment of AD [4]. Despite the emerging research on managing AD, only a small number of drugs have been approved and implemented in clinical practice. Existing therapies target a single protein/pathway and are frequently linked to adverse effects, including nausea, vomiting, diarrhea, abdominal pain, dizziness, insomnia, and weight loss [5]. AD has a complex pathology that includes Aβ aggregation, formation of neurofibrillary tangles, inflammation, and oxidative stress; thus, new approaches are required to treat AD. Herbal medicines have been used for a long time and are considered safe and effective, but they lack sufficient scientific evidence. Several types of herb-derived natural compounds such as polyphenolics, terpenoids, and flavonoids having holistic mode of action with lower side effects are being investigated for their therapeutic use in AD [6, 7].

*Piper betle*, belonging to the Piperaceae family, is commonly cultivated for its leaves which are used as a mouth freshener in various Southeast Asian countries. Ayurveda describes the analgesic and cooling properties of *P. betle* and is recommended for treating headaches, nervous pains, and nervous exhaustion [8, 9]. Previous studies have shown the antidepressant and anti-AD potential of *P. betle* [10–12]. Hydroxychavicol (HC), a bioactive component of the *P. betle* plant, is a polyphenolic compound used for its unique pharmacological properties [13, 14]. HC has shown therapeutic effects such as ROS scavenging, xanthine oxidase inhibition, antioxidant, anticancer, anti-inflammatory, and antiacetylcholinesterase activity [13, 15–20]. HC has also mitigated the streptozotocin-induced cognitive dysfunction in AD rat models and reduced the elevated levels of proinflammatory biomarkers. The study revealed that HC inhibited the activity of both β and γ-secretase enzymes without any potential side effects [20]. Despite significant studies showing the biological activity of HC against AD, the underlying mechanism is still unclear. Network pharmacology analysis using bioinformatic tools and datasets from various databases has been employed for drug development to form a network between multiple targets, multiple effects, and complex diseases [21]. The network pharmacology provides a better understanding of the interaction between active compounds and the disease targets and establishes a multilevel drug-disease target-pathway map [22]. Modern drug discovery methods utilize methods such as molecular docking and molecular dynamics (MD) simulation for virtual analysis of the binding between the protein and the drug/ligand. Molecular docking is useful for predicting the interaction force between the receptor and ligand as well as predicting the binding mode and affinity. The MD simulations give an enhanced simulation of the ligand motion within the receptor site using the principles of Newtonian mechanics, providing information about the stability and flexibility of interaction [23–25].

**Figure 1:**
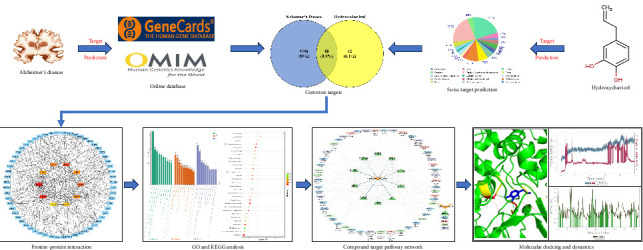
A representative scheme of the study design that shows step by step combined multiple approaches used to conduct the study.

The present study aims to perform the in silico methods to identify therapeutic targets of HC for AD, integrating target prediction, protein–protein interaction (PPI) mapping, Gene Ontology (GO) and KEGG analyses, docking studies, and MD simulations to assess target stability (Figure 1).

## 2. Materials and Methods

### 2.1. Structure Retrieval

The structure of HC was retrieved from the PubChem database (pubchem.ncbi.nlm.nih.gov) by searching the keyword “Hydroxychavicol”. The structure of was downloaded in different formats like SMILES, 2D and, 3D sdf format. The pdb structure file of HC was obtained by converting 3D sdf format to pdb format using open babel online (https://www.cheminfo.org/Chemistry/Cheminformatics/FormatConverter/index.html).

### 2.2. ADMET Analysis of HC

The ADMET analysis and drug-likeness properties of HC were done using Swiss-ADME server (https://www.swissadme.ch/). The SMILES format of HC was uploaded to the Swiss-ADME server (https://www.swissadme.ch/). The *Brain Or IntestinaL EstimateD* Prediction (BOILED-Egg) method was used for the prediction of GI absorption based on the polar and lipophilic character of HC.

### 2.3. Target Prediction

The target prediction was carried out using Swiss Target Prediction (https://www.swisstargetprediction.ch/) and Herbal Ingredients' Targets (HIT) database Version 2 (https://hit2.badd-cao.net) by either submitting SMILES or searching the keyword “Hydroxychavicol”. All the targets obtained were combined, and duplicates were removed using Microsoft Excel 2021 and standardized by the UniProt database (https://www.uniprot.org/), which was set for the human species.

### 2.4. Screening of Disease-Related Targets

The targets associated with AD were derived by searching the keyword “Alzheimer's diseases” in the DisGeNET database (https://www.disgenet.org/) and GeneCards: The Human Gene Database (https://www.genecards.org/). All the targets were combined and the duplicates were removed using Microsoft Excel 2021 and standardized by the UniProt database (https://www.uniprot.org/), which was set for the human species.

### 2.5. Mapping Common Targets

The online tool Venny 2.1 mapping platform (https://bioinfogp.cnb.csic.es/tools/venny/index.html) was used to find the interacting targets between “Alzheimer's disease” targets and “Hydroxychavicol” targets. The input provided were the gene symbols of protein targets of both HC and AD obtained from the previous sections.

### 2.6. Common Target PPI Network Construction

The intersection targets were imported into the STRING database (https://string-db.org/cgi/input). A confidence of ≥ 0.4 was taken and the free nodes were hidden to construct the PPI network. This PPI network was further processed by Cytoscape 3.7.2 software to realize visualization and screen out the core targets [26].

### 2.7. GO and KEGG Enrichment Analysis

To study the possible biological pathways of the constituents and relations between the targets, GO (https://www.geneontology.org/) and KEGG (https://www.genome.jp/kegg) analysis through the Database of Annotation, Visualization, and Integrated Discovery (DAVID) (https://david.ncifcrf.gov/tools.jsp) were performed. The species selection was set at “*Homo sapiens*”. Further analysis of the biological processes (BP), cellular component (CC), molecular function (MF), and signal pathway was done through the bioinformatic online platform (https://www.bioinformatics.com.cn).

### 2.8. Constructing the Compound Target Pathway Network

A component-target-pathway network was constructed using Cytoscape 3.7.2 software. This network, incorporating HC, core targets, and key signaling pathways, was utilized to systematically analyze the potential mechanisms of action for HC in the treatment of AD.

### 2.9. Docking of HC With Core Target Molecules

Molecular docking was carried out between HC and the hub genes using the AutoDock Vina software [27]. The three-dimensional (3D) crystal structures of all the hub genes were retrieved from the RCSB PDB databank (https://www.rcsb.org/). The pdb structure file of HC (PubChem CID 70775) was obtained by converting 3D sdf format. Then, it was used for ligand preparation in AutoDock v4.2.6 installed with the MGLTools v1.5.6 plugin and later saved in the pdbqt format. The protein structures were cleaned by deleting attached ligands, ions, and water molecules using AutoDock v4.2.6. Protein preparation was carried out using the AutoDock v4.2.6 installed with the MGLTools v1.5.6 plugin. Polar hydrogens were added to the crystal structure, charges were calculated, and the structure was saved in pdbqt format. The grid box was generated using AutoDock v4.2.6, which covered the whole protein. Keeping all the parameters as default, the docking was performed using AutoDock Vina v2.0.21. Interactions were analyzed using the BIOVIA Discovery Studio visualizer v21.1.0.20298.

### 2.10. MD Simulation

MD simulation is widely used in drug discovery for the prediction of atomic movements of proteins and complexes over a short duration based on the existing models [28]. In the current study, MD simulation was carried out for the HC-catechol-O-methyltransferase (COMT), glyceraldehyde-3-phosphate dehydrogenase (GAPDH), and Heat Shock Protein 90 Alpha Family Class A Member 1 (HSP90AA1) using Desmond 2021-4 on an Acer workstation installed with Ubuntu 22.04. OPLS-2005 Force field was used for topology generation. The complexes were prepared using the system builder platform by solvation with the simple point-charge (SPC) explicit water model in the orthorhombic simulation box. To mimic the physiological conditions, the solvated complex system was neutralized with a suitable number of Na^+^/Cl^−^ counter ions and a salt concentration of 0.15 M. The receptor-ligand complexes system was designated with the OPLS-2005 force field, and an explicit solvent model with the SPC water molecules was used in this system in the orthorhombic box. Desmond minimization of systems was performed for 100 ps and the systems were relaxed using default protocol and then the simulations were carried out at 300 K temperature and 1.0325 bar pressure for 100 ns [29].

## 3. Results

### 3.1. Structure of HC

**Figure 2:**
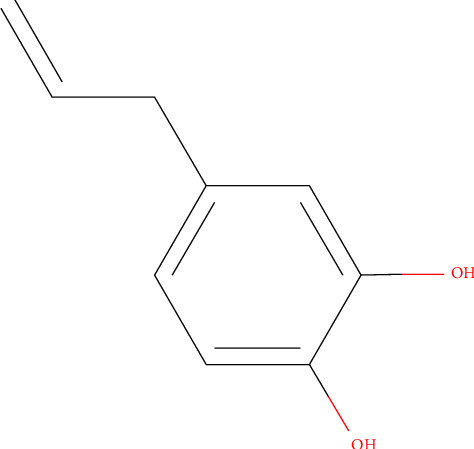
Structure of HC obtained from PubChem database.

The structure of HC was downloaded from the PubChem database under PubChem CID 70775.  Figure 2 represents the 2D structure of HC obtained from the PubChem database. The 3D structure of HC was converted from sdf format to pdb format for further in silico analysis using the Open Babel software available online (https://www.cheminfo.org/Chemistry/Cheminformatics/FormatConverter/index.html).

### 3.2. Analysis of Druglikeness

**Figure 3:**
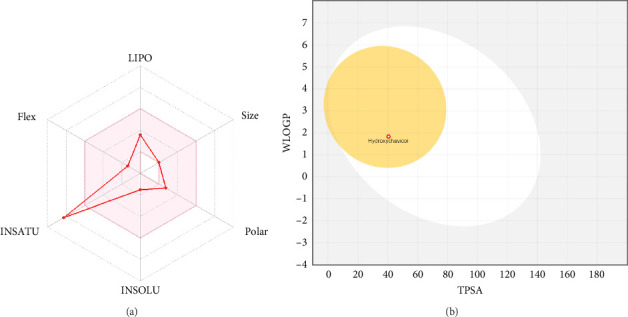
Swiss-ADME analysis of HC showing (a) oral bioavailability and (b) boiled egg map.

The druglikeliness of HC was assessed using Lipinski's rule of five, with swissADME analysis confirming that HC meets all criteria without any violations. A bioavailability score of 0.55 and high gastrointestinal absorption further support its potential as a drug-like candidate (Table 1).  Figure 3 illustrates the oral bioavailability and the boiled egg map of HC, confirming its high gastrointestinal (GI) absorption and suitability for oral administration.

### 3.3. Prediction of the HC Target Genes

**Figure 4:**
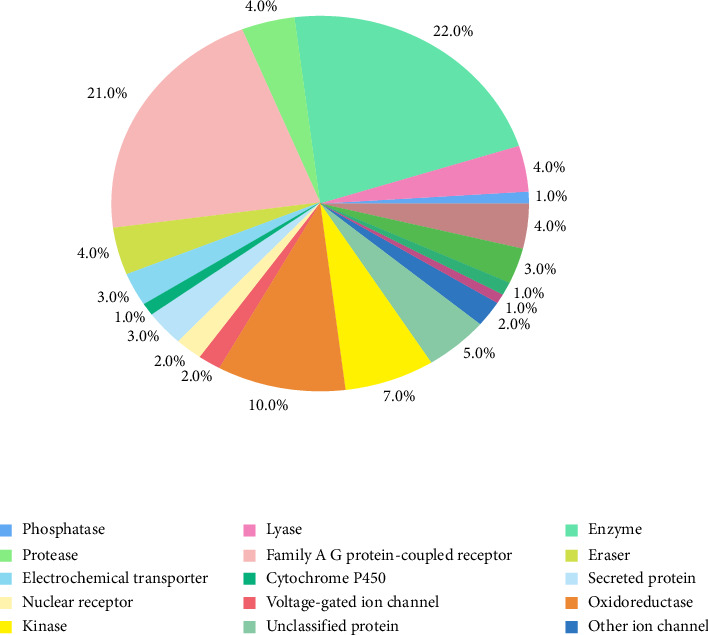
Functional classification of the proteins interacting with HC.

Based on the structure of HC, Swiss Target Prediction and the HIT database (Vsersion 2.0) were utilized to identify potential target genes, yielding a total of 100 candidates. The functional classification of these target genes, based on biochemical criteria, is presented in Figure 4. These targets primarily include family A/G protein-coupled receptors, enzymes, kinases, and proteases.

### 3.4. Identification of AD-Related Targets

The targets associated with AD were obtained from the DisGeNET database and the GeneCards database, where a total of 9794 targets were obtained.

### 3.5. Common Targets for Diseases and Drugs

**Figure 5:**
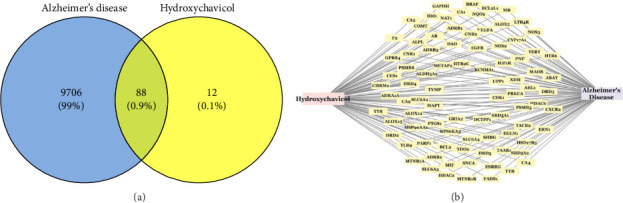
Common targets of HC and AD showing (a) venn diagram with the number of common targets and (b) the network showing those targets.

A total of 9794 AD-related targets and 100 potential target genes of HC were imported to the Venny online mapping platform. After mapping, 88 intersection targets of AD and HC were obtained (Figure 5(a)). A network was constructed showing interactive HC-target gene-AD (Figure 5(b)), which indicated that HC might affect AD by stimulating or inhibiting these target genes.

### 3.6. PPI

**Figure 6:**
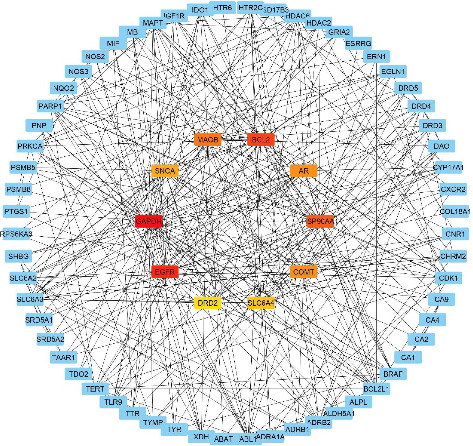
PPI networks of common target genes of HC and AD. Nodes represent the common protein targets and the lines connecting the nodes represent the interaction between two nodes. Nodes in dark color (yellow to red) represents the key hub proteins.

All common gene lists were uploaded to the STRING database, yielding 373 interaction pairs within the PPI network.  Figure 6 presents the PPI interaction map of these common genes, while Table 2 lists the top 10 hub genes. The top 10 proteins with the highest degrees of interaction were GAPDH (degree = 42), EGFR (degree = 29), BCL2 (degree = 28), HSP90AA1 (degree = 24), MAOB (degree = 22), AR (degree = 20), COMT (degree = 20), SNCA (degree = 18), SLC6A4 (degree = 17), and DRD2 (degree = 16). These were identified as the key hub proteins.

### 3.7. GO and KEGG Enrichment Analysis

**Figure 7:**
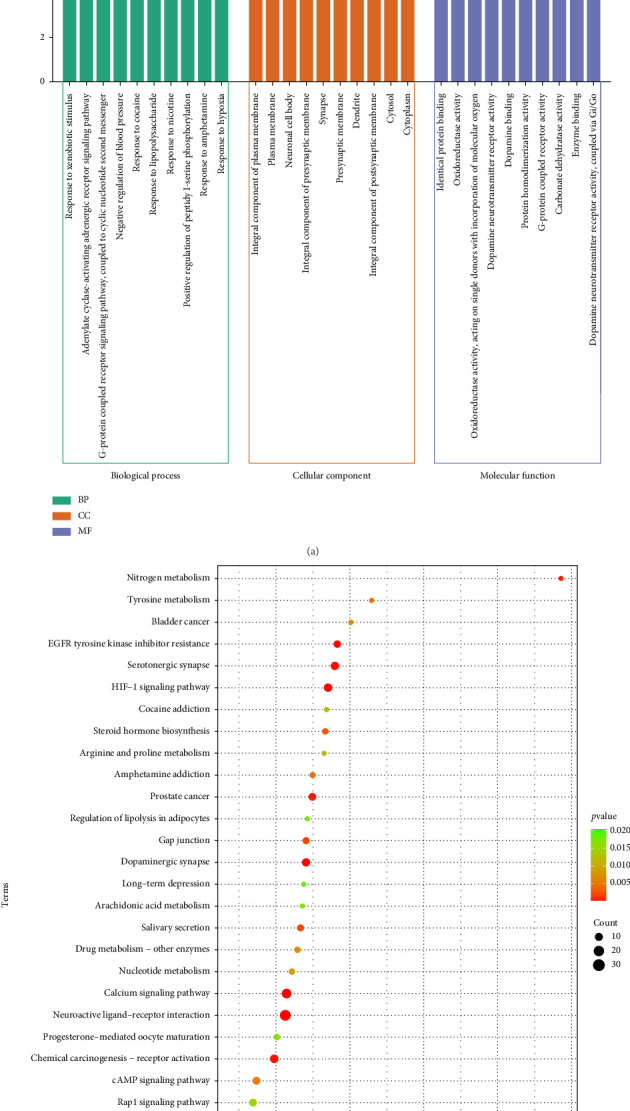
Diagram showing the GO functional and KEGG pathway enrichment analyses. (a) Top 10 enriched BP, CC, and MF terms from GO functional enrichment analysis and (b) Top 30 enriched pathways from KEGG pathway enrichment analysis.

The GO functional annotation and KEGG pathway analyses were conducted to explore the potential functions and mechanisms of the 88 common target genes. GO analysis identified a total of 206 BP, 67 MF, and 43 CC terms.  Figure 7(a) and Table 3 present the top 10 pathways from each functional category (BP, MF, and CC). Based on KEGG functional analysis, a total of 52 unique terms were obtained and Figure 7(b), and Table 4 shows the top 30 KEGG pathways. Neuroactive ligand-receptor interaction, calcium signaling pathway, serotonergic synapse, HIF-1 signaling pathway, metabolic pathways, dopaminergic synapse, EGFR tyrosine kinase inhibitor resistance, chemical carcinogenesis-receptor activation, and prostate cancer are some important pathways obtained in the KEGG analysis.

### 3.8. Compound-Target-Pathway Network

**Figure 8:**
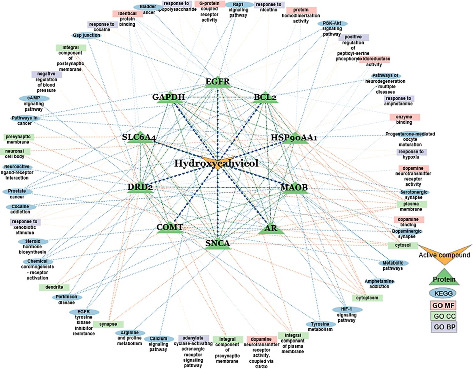
Comprehensive network of compound target protein and pathway interaction map, highlighting the key interactions between the compound, target genes, and pathways.

The compound-target-pathway network was constructed using Cytoscape by selecting the top 10 hub genes and their associated pathways. A total of 9 BP terms, 10 CC terms, 8 MF terms, and 23 KEGG terms were identified, which were interacting with the top 10 hub genes (Figure 8).

### 3.9. Molecular Docking Verification of Potential Targets

**Figure 9:**
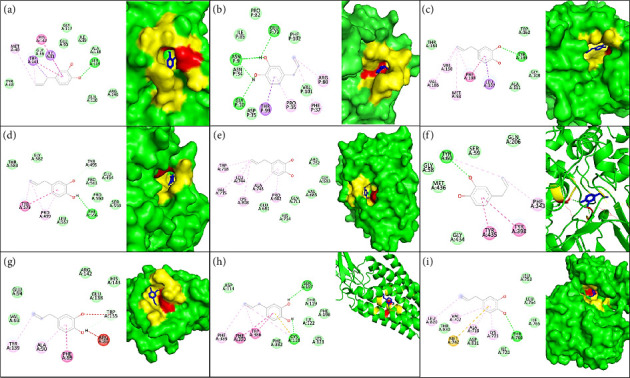
Docking of HC with top 10 hub genes intermolecular interactions and surface interaction of HC shown in blue, polar interaction shown in red, and nonpolar interaction shown in yellow. (a) COMT; (b) GAPDH; (c) HSP90AA1; (d) SLC6A4; (e) AR; (f) MAOB; (g) BCL2; (h) DRD2; and (i) EGFR.

Further validation of the potential targets through molecular docking was carried out to evaluate the reliability of the anti-AD targets of HC. The docking analysis was done with the top 10 genes. The interaction analysis of HC with hub genes revealed the free binding energies ranging from −5.6 to −8.9 kcal/mol. Among these, COMT, GAPDH, and HSP90AA1 emerged as the top three targets based on their binding affinities of −8.9 kcal/mol, −6.7 kcal/mol, and −6.5 kcal/mol, respectively. HC was found to interact with several key active site residues within COMT, GAPDH, and HSP90AA1, as detailed in Table 5 and Figure 9.

HC was found to bind with catechol-O-methyltransferase (COMT, PDB ID: 6I3D), where Ser-119 formed a hydrogen bond, and nonpolar interactions were observed with Ile-91, Trp-143, Met-40, and His-142 (Figure 9(a)). The binding site of HC on glyceraldehyde-3-phosphate dehydrogenase (GAPDH, PDB ID: 1U8F) was located within a pocket surrounded by Asn-9, Gly-10, Asn-34, Pro-36, Phe-37, Glu-79, Arg-80, and Thr-99 (Figure 9(b)). HC formed hydrogen bonds with Asn-9, Gly-10, Asn-34, and Glu-79, while nonpolar/hydrophobic interactions were observed with Arg-80, Phe-37, Pro-36, and Thr-99. Docking analysis of HC with heat shock protein 90 alpha family class A member 1 (HSP90AA1, PDB ID: 4BQG) revealed a hydrogen bond interaction with Tyr-139, along with hydrophobic interactions involving Met-98, Leu-107, Phe-138, Val-150, and Val-186 (Figure 9(c)).

The docking of HC with solute carrier family 6 member 4 (SLC6A4, PDB ID- 5I6X) achieved a binding score of −6.4 kcal/mol with stable hydrogen bonding interaction with Phe-556 and Tyr-495. In addition, hydrophobic interactions with Pro-499 and Tyr-579 further contribute to the stability (Figure 9(d)). The docking of HC with androgen receptor (AR, PDB ID 4OEA) yielded a docking score of −6.3 kcal/mol with hydrogen bond interaction (Gln-711 and Arg-752) and hydrophobic interaction (Gln-711, Trp-718, Leu-744, Ala-748, and Lys-808) (Figure 9(e)). The interaction study of HC with monoamine oxidase B (MAOB, PDB ID-1S2Q) showed hydrogen bonding at Tyr60 and hydrophobic interaction at Tyr-435, Tyr-398, and Phe-343 with a docking score of −6.2 kcal/mol (Figure 9(f)).

Similarly, HC was observed to interact with other key hub genes, including B-cell lymphoma 2 (BCL2, PDB ID: 2 W3L) (Figure 9(g)), dopamine receptor D2 (DRD2, PDB ID: 7DFP) (Figure 9(h)), and epidermal growth factor receptor (EGFR, PDB ID: 1M17) (Figure 9(i)). The corresponding binding energies and interacting residues are detailed in Table 5. Since the SNCA is an intrinsically disordered protein, it is difficult to target due to its lack of defined small-molecule binding pockets, so the docking between SNCA and HC was unsuccessful [30].

### 3.10. Dynamics Simulations

**Figure 10:**
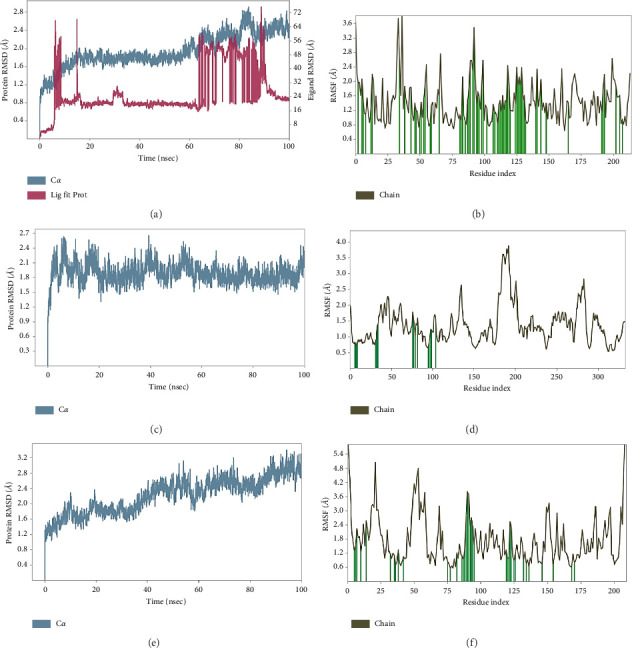
RMSD of protein backbone versus time and RMSF of individual amino acids of (a and b) COMT domain (PDB Code: 6I3D); (c and d) GAPDH (PDB Code: 1U8F); and (e and f) HSP90AA1 (PDB Code: 4BQG) bound with HC.

The highest binding affinity (kcal/mol) scorer ligand-protein complexes, that is, HC- COMT, GAPDH, and HSP90AA1 were selected for further MD simulation study. Root mean square deviation (RMSD), is a measure of the average displacement shift between a group of atoms in a given frame and a reference frame. A lower RMSD value indicates that the ligand is more stable because it is in better alignment with the binding pocket (Figure 10).

**Figure 11:**
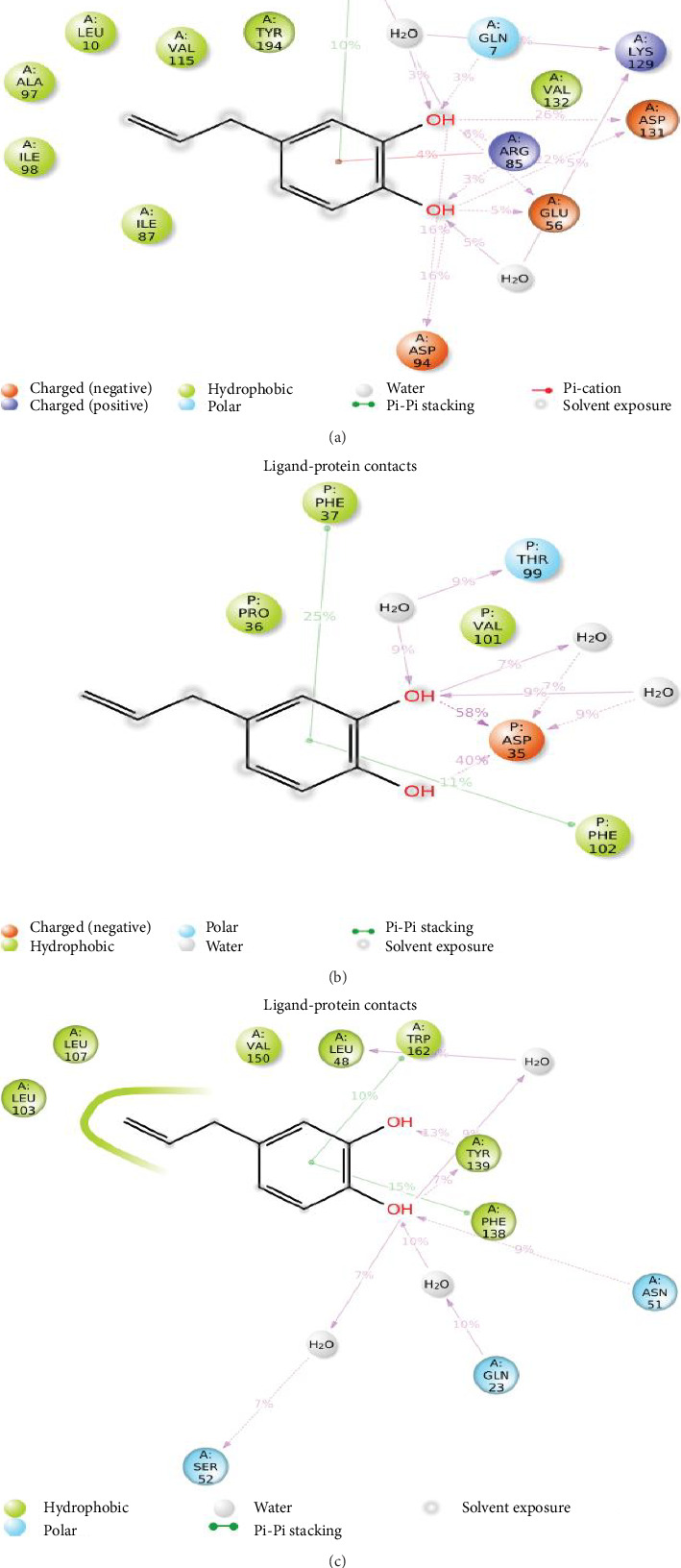
Post dynamics interaction of HC on protein residues of (a) COMT domain (PDB Code: 6I3D); (b) GAPDH (PDB Code: 1U8F); and (c) HSP90AA1 (PDB Code: 4BQG).

The difference between the maximum and minimum from the average was calculated as 0.343 and 0.642 (Figure 10). The lower difference suggests that the protein-ligand combination is more stable. The RMSD value slowly fluctuated up to 15 ns and then sustained for 35 ns; after that, it increased slightly after 80 ns for the HC-COMT complex. For the HC-GAPDH complex, it remained constant after 20 ns which confirmed that the system is equilibrated. The difference between atomic locations, or RMSF, is shown versus the number of residues. Figure 10 shows that Ser-119 of COMT, Asn-9, Gly-10 Asn-34, Glu-79 of GAPDH, and Tyr-139 of HSP90AA1 did not fluctuate, which confirmed the involvement of these residues in the stabilization of ligand. The interaction of HC after dynamics showed that it stabilized with its targets through distinct bonding. Specifically, HC formed hydrogen bonds with Gln7, Glu56, and Asp131 of COMT (Figure 11(a)). Similarly HC formed hydrogen bonds with Asp35, along with π-π stacking interactions with Phe37 and Phe102 of GAPDH (Figure 11(b)). Furthermore, HC formed hydrogen bonds with Asn51 and Tyr139, as well as π-π stacking interactions with Phe138 and Trp162 of HSP90AA1 (Figure 11(c)).

## 4. Discussion

AD is one of the most prevalent neurological diseases, with a complex etiology, which encompasses various pathways and molecules. Existing single-target drugs have not demonstrated significant efficacy in controlling disease progression; therefore, there is a need for the development of drugs with holistic effect [31].

AD is one of the most prevalent neurological diseases, with a complex etiology and pathology, which encompasses various pathways and molecules. Currently, available single-target drugs have not shown any promising effect in controlling disease progression; therefore, there is a need for the development of new drugs [31]. The natural compounds have emerged as potential therapeutic agents, many of which have shown neuroprotective effects due to their diverse pharmacological activities and ability to target various pathological mechanisms [32]. HC, a phenolic compound found in *P. betel*, is known for its antioxidant, anti-inflammatory properties and anti-AD activity [20, 33].

This study was conducted to decipher the potential AD-related target of HC using a combination of network pharmacology, molecular docking, and dynamic simulation study. A total of 88 intersecting targets were obtained between HC and AD in which GAPDH, EGFR, BCL2, HSP90AA1, MAOB, AR, COMT, SNCA, SLC6A4, and DRD2 were the top 10 hub genes. The proteins encoded by these top hub genes were analyzed as a target of HC through molecular docking studies. Then, the dynamics simulation analysis of HC with the top three proteins having the highest binding energy was conducted. Docking analysis results confirmed stable binding and interaction of HC with COMT, GAPDH, and HSP90AA1, where the binding energy of −8.9, −6.7, and −6.5 kcal/mol, respectively, were observed. These targets are associated with neurophysiology and AD pathogenesis through various mechanisms. COMT catalyzes the degradation of catecholamine neurotransmitters such as dopamine, noradrenaline, and adrenaline by the transfer of methyl group from S-adenosyl-methionine (SAM) to a hydroxyl group on a catechol nucleus. COMT plays a significant role in regulating the dopamine levels in the prefrontal cortex, thus playing a crucial role in learning and memory. Impairment of the COMT is associated with rapid progression of AD and disease severity [34, 35]. The COMT gene, particularly the rs4680 polymorphism, has been linked to AD [36]. HC interacts with important residues of COMT such as Trp143, His142, and Met40. As per previous reports, Trp143 and His 142 forms van der Waals interactions with SAM, and Met40 lies close to transferring methyl group of COMT [37, 38].

GAPDH is an abundantly expressed protein that is mainly involved in the glucose metabolism. Several studies have shown the direct or indirect involvement of GAPDH in AD pathology, and its interaction with amyloid precursor protein and NFTs has also been reported. GAPDH is subjected to oxidative modification in the AD brain, leading to inhibited dehydrogenase activity. This modification contributes to neurodegeneration by affecting neuronal cell development and survival [39]. GAPDH has also been reported as the disease-susceptible gene in a systematic meta-analysis of the polymorphism in AD [40]. The HC forms hydrogen bonds with Asn-9, Gly-10, Asn-34, and Glu-79 and forms hydrophobic interactions with Arg-80, Phe-37, Pro-36, and Thr-99 of GAPDH, which lies close to the NAD^+^ binding site of GAPDH. The amino acid residues Pro36, Phe37, Thr99, and Phe102 form the hydrophobic interactions with the adenine group of NAD^+^ [41].

HSP90 is a ubiquitously and highly expressed chaperone protein involved in protein folding and transport. HSP90 also regulates tau metabolism and Aβ processing. There are two major isoforms of HSP90: HSP90α (HSP90AA1) and HSP90β (HSP90AB1) [42]. Expression of both these isoforms are downregulated in the AD [43]. The HC binds near the ATP binding site of HSP90AA1 and forms the hydrogen bond with Tyr139 and hydrophobic interactions with Val150, Val186, Met98, Phe138, and Leu107. The Phe138 makes the hydrogen bond with the nitro group of ATP [44]. Previous studies have highlighted the potential of COMT and HSP90 inhibitors as promising therapeutic candidates for AD. However, further research is needed to fully explore their efficacy, safety, and feasibility for drug development [35, 45–48].

Our study highlights the potential role of HC in AD treatment by affecting important signaling molecules such as HIF-1, cAMP, Rap1, PI3K-Akt, steroid hormone biosynthesis, neuroactive ligand-receptor interaction, calcium signaling, tyrosine metabolism, arginine and proline metabolism, and dopaminergic synapse pathways. The role of the PI3K/Akt/mTOR signaling in the progression of AD mainly depends on its regulation and is generally downregulated in AD [49]. The PI3K-Akt activation is induced by the phosphorylation of mTOR, which may have a positive effect on synaptic plasticity and memory formation [50]. The PI3K-Akt-mTOR pathways also play an important role in maintaining the balance between the autophagy and the protein synthesis [51, 52]. mTORC2 phosphorylates Akt which in turn is an activator of mTORC1, inhibiting autophagy and promoting protein synthesis [53]. A previous study shows that cotreatment of HC and curcumin increased phosphorylation of mTOR and MAPK, thus activating them in different leukemic cell lines, including K562, KCL22, and Molt4 [54]. The Akt signaling also downregulates glycogen synthase kinase-3β (GSK-3β), CREB, and Tau in the neurons, thereby playing a vital role in AD pathology [55].

The cAMP signaling pathway and CERB are important transcription regulators involved in neuronal health, synaptic plasticity, and memory formation [56]. The increased CSF cAMP levels have been observed in AD patients, and a significant correlation between cAMP and tau protein levels in CSF has been observed [57]. The ethanolic extract of *P. betle* leaves has prevented the synthesis of the cAMP, thus reducing the CREB phosphorylation in human melanoma, MNT-1 cell line [58]. The calcium and RAP1 signaling are involved in controlling important functions in brain. RAP1 is engaged in processes such as neuron excitation, synaptic plasticity, gene expression, and maintaining the membrane through calcium-dependent activation and ERK signaling [59]. The HIF1 signaling pathway activates various important genes in response to cellular hypoxia, such as VEGF, erythropoietin, and iNOS. HIF-1α promotes AD pathology by activating β/γ-secretases and thereby increasing the Aβ generation and neuroinflammation [60].

## 5. Conclusion

Overall, this study identified proteins such as COMT, GAPDH, and HSP90AA1, along with signaling molecules such as HIF-1, cAMP, Rap1, and PI3K-Akt, as significant therapeutic targets of HC. Currently, there are no therapeutic agents approved by the U.S. Food and Drug Administration (FDA) that specifically target COMT, GAPDH, and HSP90AA1 for the treatment of AD. The findings from this study, in conjunction with ongoing research, pave the way for exploring selective inhibitors of these targets as potential therapeutic strategies for preventing and treating AD.

## Figures and Tables

**Table 1:** Molecular properties of HC to determine druglikeliness.

S. no.	Property	Value
1	Molecular weight	150.17 g/mol
2	XLogP3-AA	0.77
3	TPSA	40.46 Å^2^
4	Rotatable bonds	2
5	H-bonds donor	2
6	H-bonds acceptor	2
7	Molar refractivity	44.59
8	Bioavailability score	0.55
9	Water solubility	Very soluble
10	GI absorption	High
11	Blood blood–brain barrier permeability	Yes

**Table 2:** Top 10 genes obtained from the PPI interaction map.

S. No.	Node	Protein name	Maximal correlation coefficient (MCC)	Degree
1	GAPDH	Glyceraldehyde-3-phosphate dehydrogenase (GAPDH)	863,338	42
2	EGFR	Epidermal growth factor receptor (EGFR)	863,059	29
3	BCL2	B-cell lymphoma 2 (BCL2)	863,169	28
4	HSP90AA1	Heat shock protein 90 alpha family class a member 1 (HSP90AA1)	862,097	24
5	MAOB	Monoamine oxidase B (MAOB)	12,474	22
6	AR	Androgen receptor (AR)	846,799	20
7	COMT	Catechol-O-methyltransferase (COMT)	12,875	20
8	SNCA	Synuclein alpha (SNCA)	10,318	18
9	SLC6A4	Solute carrier family 6 member 4 (SCL6A4)	11,109	17
10	DRD2	Dopamine receptor D2 (DRD2)	12,434	16

**Table 3:** Top 10 enriched BP, CC, and MF terms from GO functional enrichment analysis.

Ontology	Term	Description	Count
BP	GO:0009410	Response to xenobiotic stimulus	18
BP	GO:0071880	Adenylate cyclase-activating adrenergic receptor signaling pathway	7
BP	GO:0007187	G-protein coupled receptor signaling pathway, coupled to cyclic nucleotide second messenger	9
BP	GO:0045776	Negative regulation of blood pressure	7
BP	GO:0042220	Response to cocaine	7
BP	GO:0032496	Response to lipopolysaccharide	10
BP	GO:0035094	Response to nicotine	7
BP	GO:0033138	Positive regulation of peptidyl–serine phosphorylation	8
BP	GO:0001975	Response to amphetamine	6
BP	GO:0001666	Response to hypoxia	9
CC	GO:0005887	Integral component of plasma membrane	25
CC	GO:0005886	Plasma membrane	48
CC	GO:0043025	Neuronal cell body	12
CC	GO:0099056	Integral component of presynaptic membrane	6
CC	GO:0045202	Synapse	12
CC	GO:0042734	Presynaptic membrane	7
CC	GO:0030425	Dendrite	11
CC	GO:0099055	Integral component of postsynaptic membrane	5
CC	GO:0005829	Cytosol	42
CC	GO:0005737	Cytoplasm	41
MF	GO:0042802	Identical protein binding	32
MF	GO:0016491	Oxidoreductase activity	13
MF	GO:0016702	Oxidoreductase activity, acting on single donors with incorporation of molecular oxygen, incorporation of two atoms of oxygen	5
MF	GO:0004952	Dopamine neurotransmitter receptor activity	4
MF	GO:0035240	Dopamine binding	4
MF	GO:0042803	Protein homodimerization activity	15
MF	GO:0004930	G-protein coupled receptor activity	14
MF	GO:0004089	Carbonate dehydratase activity	4
MF	GO:0019899	Enzyme binding	10
MF	GO:0001591	Dopamine neurotransmitter receptor activity, coupled via Gi/Go	3

**Table 4:** Top 30 enriched terms from the KEGG pathway enrichment analysis.

S. No.	Term	Pathway	Count	*p* value
1	hsa04080	Neuroactive ligand-receptor interaction	20	1.03E-09
2	hsa04020	Calcium signaling pathway	14	6.85E-07
3	hsa04726	Serotonergic synapse	10	1.32E-06
4	hsa04066	HIF-1 signaling pathway	9	8.61E-06
5	hsa01100	Metabolic pathways	32	1.59E-05
6	hsa04728	Dopaminergic synapse	9	3.50E-05
7	hsa01521	EGFR tyrosine kinase inhibitor resistance	7	1.01E-04
8	hsa05207	Chemical carcinogenesis-receptor activation	10	1.79E-04
9	hsa05215	Prostate cancer	7	3.15E-04
10	hsa05200	Pathways in cancer	15	4.53E-04
11	hsa00910	Nitrogen metabolism	4	5.21E-04
12	hsa04540	Gap junction	6	0.001492
13	hsa04970	Salivary secretion	6	0.001907
14	hsa00140	Steroid hormone biosynthesis	5	0.002849
15	hsa05031	Amphetamine addiction	5	0.004198
16	hsa00350	Tyrosine metabolism	4	0.004801
17	hsa04024	cAMP signaling pathway	8	0.005603
18	hsa05219	Bladder cancer	4	0.006927
19	hsa00983	Drug metabolism: other enzymes	5	0.007096
20	hsa01232	Nucleotide metabolism	5	0.008765
21	hsa05030	Cocaine addiction	4	0.011336
22	hsa00330	Arginine and proline metabolism	4	0.011978
23	hsa05012	Parkinson disease	8	0.013438
24	hsa05022	Pathways of neurodegeneration: multiple diseases	11	0.014917
25	hsa04015	Rap1 signaling pathway	7	0.015059
26	hsa04914	Progesterone-mediated oocyte maturation	5	0.016289
27	hsa04923	Regulation of lipolysis in adipocytes	4	0.017858
28	hsa04151	PI3K-Akt signaling pathway	9	0.019502
29	hsa04730	Long-term depression	4	0.019538
30	hsa00590	Arachidonic acid metabolism	4	0.02041

**Table 5:** Binding scores, interactions, and participating residues in the docking of HC with top 10 hub genes.

S. no.	Gene name	PDB ID	Free binding energy (kcal/mol)	Hydrogen bonding	Hydrophobic interaction
1	COMT	6I3D	−8.9	Ser-119	Ile-91, Trp-143, Met-40, His-142
2	GAPDH	1U8F	−6.7	Asn-9, Gly-10, Asn-34, Glu-79	Arg-80, Phe-37, Pro-36, Thr-99
3	HSP90AA1	4BQG	−6.5	Tyr-139	Val-150, Val-186, Met-98, Phe-138, Leu-107
4	SLC6A4	5I6X	−6.4	Phe-556, Tyr-495	Pro-499, Tyr-579,
5	AR	4OEA	−6.3	Gln-711, Arg-752	Gln-711, Trp-718, Leu-744, Ala-748, Lys-808
6	MAOB	1S2Q	−6.2	Tyr-60	Tyr-435, Tyr-398, Phe-343
7	BCL2	2 W3L	−5.9	Trp-135	Phe-89, Ala-90, Tyr-139
8	DRD2	7DFP	−5.9	Cys-118, Ser-197	Trp-386, Phe-389, Phe-390
9	EGFR	1M17	−5.6	Thr-766	Leu-820, Val-702, Ala719, Lys-721
10	SNCA	α-Synuclein (SNCA) is an intrinsically disordered protein (IDP) and is, therefore, difficult to target, owing to its lack of defined small-molecule binding pockets [30]			

## Data Availability

The data used to support the findings of this study are included within the article.

## References

[1] International A. D., Guerchet M., Prince M., Prina M. (2020). *Numbers of People With Dementia Worldwide: An Update to the Estimates in the World Alzheimer Report 2015*.

[2] Morgan S. L., Naderi P., Koler K. (2022). Most Pathways Can Be Related to the Pathogenesis of Alzheimer’s Disease. *Frontiers in Aging Neuroscience*.

[3] Breijyeh Z., Karaman R. (2020). Comprehensive Review on Alzheimer’s Disease: Causes and Treatment. *Molecules*.

[4] Chowdhury S., Chowdhury N. S. (2023). Novel Anti-Amyloid-Beta (Aβ) Monoclonal Antibody Lecanemab for Alzheimer’s Disease: A Systematic Review. *International Journal of Immunopathology & Pharmacology*.

[5] Atri A. (2019). Current and Future Treatments in Alzheimer’s Disease. *Seminars in Neurology*.

[6] Bukhari S. N. A. (2022). Dietary Polyphenols as Therapeutic Intervention for Alzheimer’s Disease: A Mechanistic Insight. *Antioxidants*.

[7] Rahman M. M., Rahaman M. S., Islam M. R. (2021). Role of Phenolic Compounds in Human Disease: Current Knowledge and Future Prospects. *Molecules*.

[8] Prasanna S. V., Ramya D., Haritha C., Pandy V., Nadendla R. R. (2021). A Comprehensive Review on the Therapeutic Potential of Piper Betel Leaf for the Treatment of Neurological Diseases. *International Journal of Botany Studies*.

[9] Reddy P. S., Gupta R. K., Reddy S. M. (2016). Analgesic and Anti-inflammatory Activity of Hydroalcoholic Extract of Piper Betle Leaves in Experimental Animals. *International Journal of Basic & Clinical Pharmacology*.

[10] Gulhane H., Misra A. K., Reddy P., Pandey D., Gulhane R., Varma S. K. (2015). Effects of Piper Betle Leaves (Paan) Extract as Anti-Depressant and Anti-Anxiety in Experimental Animals. *Mintage Journal of Pharmaceutical and Medical Sciences*.

[11] Meti V., Ruckmani A., Chandrashekar K. (2012). Antidepressant Activity of Ethanolic Extract of Piper Betle Leaves in Mice. *Current Research in Neuroscience*.

[12] Upadhyaya S., Gangachannaiah S., Chandrashekar P. L. (2019). Effect of Piper Betel Leaf Extract on Learning and Memory in Aluminium Chloride Induced Alzheimer’s Disease in Wistar Rats. *Biomedical and Pharmacology Journal*.

[13] Ali I., Satti N. K., Dutt P., Prasad R., Khan I. A. (2016). Hydroxychavicol: A Phytochemical Targeting Cutaneous Fungal Infections. *Scientific Reports*.

[14] Sharma S., Khan I. A., Ali I. (2009). Evaluation of the Antimicrobial, Antioxidant, and Anti-Inflammatory Activities of Hydroxychavicol for its Potential Use as an Oral Care Agent. *Antimicrobial Agents and Chemotherapy*.

[15] Chakraborty J. B., Mahato S. K., Joshi K. (2012). Expression of Concern: Hydroxychavicol, a *Piper Betle* Leaf Component, Induces Apoptosis of CML Cells Through Mitochondrial Reactive Oxygen Species-Dependent JNK and Endothelial Nitric Oxide Synthase Activation and Overrides Imatinib Resistance. *Cancer Science*.

[16] Mohamad N. A., Rahman A. A., Sheikh Abdul Kadir S. H. (2022). Hydroxychavicol as a Potential Anticancer Agent (Review). *Oncology Letters*.

[17] Murata K., Nakao K., Hirata N. (2009). Hydroxychavicol: A Potent Xanthine Oxidase Inhibitor Obtained From the Leaves of Betel, Piper Betle. *Journal of Natural Medicines*.

[18] Rajedadram A., Pin K. Y., Ling S. K., Yan S., Looi M. (2021). Hydroxychavicol, a Polyphenol From Piper Betle Leaf Extract, Induces Cell Cycle Arrest and Apoptosis in TP53-Resistant HT-29 Colon Cancer Cells. *Journal of Zhejiang University-Science B*.

[19] Debnath M., Das S., Bhowmick S., Karak S., Saha A., De B. (2021). Anti-Alzheimer’s Potential of Different Varieties of Piper Betle Leaves and Molecular Docking Analyses of Metabolites. *Free Radicals and Antioxidants*.

[20] Pandey A., Bani S. (2010). Hydroxychavicol Inhibits Immune Responses to Mitigate Cognitive Dysfunction in Rats. *Journal of Neuroimmunology*.

[21] Li S. (2021). Network Pharmacology Evaluation Method Guidance-Draft. *World Journal of Traditional Chinese Medicine*.

[22] Qi P., Li J., Gao S. (2020). Network Pharmacology-Based and Experimental Identification of the Effects of Quercetin on Alzheimer’s Disease. *Frontiers in Aging Neuroscience*.

[23] De Vivo M., Masetti M., Bottegoni G., Cavalli A. (2016). Role of Molecular Dynamics and Related Methods in Drug Discovery. *Journal of Medicinal Chemistry*.

[24] Singh S., Bani Baker Q., Singh D. B., Singh D. B., Pathak R. K. (2022). Molecular Docking and Molecular Dynamics Simulation. *Bioinformatics*.

[25] Ye J., Li L., Hu Z. (2021). Exploring the Molecular Mechanism of Action of Yinchen Wuling Powder for the Treatment of Hyperlipidemia, Using Network Pharmacology, Molecular Docking, and Molecular Dynamics Simulation. *BioMed Research International*.

[26] Rambabu M., Navanneth Gowda M., Selvam P. K. (2024). Transcriptomic Insights into Skin Cancer: A Bioinformatics and Network Biology Approach to Biomarker Identification. *Journal of King Saud University Science*.

[27] Kumar P. H., Rambabu M., Vijayakumar V., Sarveswari S. (2023). Palladium-Mediated Synthesis of 2-([Biphenyl]-4-Yloxy)quinolin-3-Carbaldehydes Through Suzuki–Miyaura Cross-Coupling and Their In Silico Breast Cancer Studies on the 3ERT Protein. *ACS Omega*.

[28] Hollingsworth S. A., Dror R. O. (2018). Molecular Dynamics Simulation for All. *Neuron*.

[29] Jnoff E., Albrecht C., Barker J. J. (2014). Binding Mode and Structure–Activity Relationships Around Direct Inhibitors of the Nrf2–Keap1 Complex. *ChemMedChem*.

[30] Zhang P., Park H. J., Zhang J. (2020). Translation of the Intrinsically Disordered Protein α-Synuclein Is Inhibited by a Small Molecule Targeting its Structured mRNA. *Proceedings of the National Academy of Sciences*.

[31] Burns S., Selman A., Sehar U., Rawat P., Reddy A., Reddy P. (2022). Therapeutics of Alzheimer’s Disease: Recent Developments. *Antioxidants*.

[32] Andrade S., Ramalho M. J., Loureiro J. A., Pereira M. D. C. (2019). Natural Compounds for Alzheimer’s Disease Therapy: A Systematic Review of Preclinical and Clinical Studies. *International Journal of Molecular Sciences*.

[33] Pin K., Abdullah L., Ahmad A. R. (2010). Antioxidant and Anti-Inflammatory Activities of Extracts of Betel Leaves (Piper Betle) From Solvents With Different Polarities. *Journal of Tropical Forest Science*.

[34] Ji Y., Shi Z., Liu M., Liu S., Liu S., Wang J. (2014). Association Between the COMT Val158Met Genotype and Alzheimer’s Disease in the Han Chinese Population. *Dementia and Geriatric Cognitive Disorders Extra*.

[35] Serretti A., Olgiati P. (2012). Catechol-o-Methyltransferase and Alzheimer’s Disease: A Review of Biological and Genetic Findings. *CNS & Neurological Disorders-Drug Targets*.

[36] Porcelli S., Calabrò M., Crisafulli C. (2019). Alzheimer’s Disease and Neurotransmission Gene Variants: Focus on Their Effects on Psychiatric Comorbidities and Inflammatory Parameters. *Neuropsychobiology*.

[37] Cruz-Vicente P., Gonçalves A. M., Ferreira O. (2021). Discovery of Small Molecules as Membrane-Bound Catechol-O-Methyltransferase Inhibitors With Interest in Parkinson’s Disease: Pharmacophore Modeling, Molecular Docking and In Vitro Experimental Validation Studies. *Pharmaceuticals*.

[38] Czarnota S., Johannissen L. O., Baxter N. J. (2019). Equatorial Active Site Compaction and Electrostatic Reorganization in Catechol-O-Methyltransferase. *ACS Catalysis*.

[39] Butterfield D. A., Hardas S. S., Lange M. (2010). Oxidatively Modified Glyceraldehyde-3-Phosphate Dehydrogenase (GAPDH) and Alzheimer’s Disease: Many Pathways to Neurodegeneration. *Journal of Alzheimer’s Disease*.

[40] Bertram L., McQueen M. B., Mullin K., Blacker D., Tanzi R. (2007). Systematic Meta-Analyses of Alzheimer Disease Genetic Association Studies: The AlzGene Database. *Nature Genetics*.

[41] Jenkins J. L., Tanner J. J. (2006). High-Resolution Structure of Human D-Glyceraldehyde-3-Phosphate Dehydrogenase. *Acta Crystallographica Section D Biological Crystallography*.

[42] Ou J. R., Tan M. S., Xie A. M., Yu J. T., Tan L. (2014). Heat Shock Protein 90 in Alzheimer’s Disease. *BioMed Research International*.

[43] Gonzalez-Rodriguez M., Villar-Conde S., Astillero-Lopez V. (2021). Neurodegeneration and Astrogliosis in the Human CA1 Hippocampal Subfield are Related to Hsp90ab1 and Bag3 in Alzheimer’s Disease. *International Journal of Molecular Sciences*.

[44] Brasca M. G., Mantegani S., Amboldi N. (2013). Discovery of NMS-E973 as Novel, Selective and Potent Inhibitor of Heat Shock Protein 90 (Hsp90). *Bioorganic & Medicinal Chemistry*.

[45] Alam Q., Alam M. Z., Sait K. H. W. (2017). Translational Shift of HSP90 as a Novel Therapeutic Target From Cancer to Neurodegenerative Disorders: An Emerging Trend in the Cure of Alzheimer’s and Parkinson’s Diseases. *Current Drug Metabolism*.

[46] Blair L. J., Sabbagh J. J., Dickey C. A. (2014). Targeting Hsp90 and its Co-Chaperones to Treat Alzheimer’s Disease. *Expert Opinion on Therapeutic Targets*.

[47] Patel C. N., Georrge J. J., Modi K. M. (2018). Pharmacophore-Based Virtual Screening of Catechol-O-Methyltransferase (COMT) Inhibitors to Combat Alzheimer’s Disease. *Journal of Biomolecular Structure and Dynamics*.

[48] Ansar S., Burlison J. A., Hadden M. K. (2007). A Non-Toxic Hsp90 Inhibitor Protects Neurons From Aβ-Induced Toxicity. *Bioorganic & Medicinal Chemistry Letters*.

[49] Razani E., Pourbagheri-Sigaroodi A., Safaroghli-Azar A., Zoghi A., Shanaki-Bavarsad M., Bashash D. (2021). The PI3K/Akt Signaling Axis in Alzheimer’s Disease: A Valuable Target to Stimulate or Suppress?. *Cell Stress & Chaperones*.

[50] Yi J. H., Baek S. J., Heo S. (2018). Direct Pharmacological Akt Activation Rescues Alzheimer’s Disease Like Memory Impairments and Aberrant Synaptic Plasticity. *Neuropharmacology*.

[51] Liu Y., Liu F., Grundke‐Iqbal I., Iqbal K., Gong C. (2011). Deficient Brain Insulin Signalling Pathway in Alzheimer’s Disease and Diabetes. *The Journal of Pathology*.

[52] Steen E., Terry B. M., J Rivera E. (2005). Impaired Insulin and Insulin-Like Growth Factor Expression and Signaling Mechanisms in Alzheimer’s Disease – Is This Type 3 Diabetes?. *Journal of Alzheimer’s Disease*.

[53] Gabbouj S., Ryhänen S., Marttinen M. (2019). Altered Insulin Signaling in Alzheimer’s Disease Brain – Special Emphasis on PI3K-Akt Pathway. *Frontiers in Neuroscience*.

[54] Chaudhuri J., Chowdhury A. A., Biswas N. (2014). Superoxide Activates mTOR-eIF4E-Bax Route to Induce Enhanced Apoptosis in Leukemic Cells. *Apoptosis*.

[55] Limantoro J., de Liyis B. G., Sutedja J. C. (2023). Akt Signaling Pathway: A Potential Therapy for Alzheimer’s Disease Through Glycogen Synthase Kinase 3 Beta Inhibition. *The Egyptian Journal of Neurology, Psychiatry and Neurosurgery*.

[56] Sharma V. K., Singh T. G. (2021). CREB: A Multifaceted Target for Alzheimer’s Disease. *Current Alzheimer Research*.

[57] Martínez M., Fernández E., Frank A., Guaza C., de la Fuente M., Hernanz A. (1999). Increased Cerebrospinal Fluid cAMP Levels in Alzheimer’s Disease. *Brain Research*.

[58] Alam B., Akter F., Parvin N. (2013). Antioxidant, Analgesic and Anti-Inflammatory Activities of the Methanolic Extract of Piper Betle Leaves. *Avicenna Journal of Phytomedicine*.

[59] Kosuru R., Chrzanowska M. (2020). Integration of Rap1 and Calcium Signaling. *International Journal of Molecular Sciences*.

[60] Wang Y.-Y., Huang Z.-T., Yuan M.-H. (2021). Role of Hypoxia Inducible Factor-1α in Alzheimer’s Disease. *Journal of Alzheimer’s Disease*.

